# 

**Published:** 1790

**Authors:** 


### CONTENTS.

Page

I. *A Case of Suppression of Urine in which Relief was obtained from puncturing the Bladder above the Pubis, but which afterwards terminated fatally; with an Account of the Appearances on Dissection; and some Remarks on the Paracentesis of the Bladder. By Mr.* Francis Turner, *Surgeon at Yarmouth.* — — — — 7

II. *Case of a schirrous Affection of the Stomach; with an Account of the Appearances on Dissection. By Mr.* William Loftie, *Surgeon at Canterbury.* — — 17

III. *Farther Account of a new Method of treating Diseases of the Joints of the Knee and Elbow. By Mr.* H. Park, *one of the Surgeons of the Liverpool Infirmary.* — 22

IV. *Observations on the Use of Electricity in Deafness. By Mr.* William Blizard, *F. R. S. and S. A. Surgeon of the London Hospital.* 31

V. *Observations on the Angustura Bark. By* Augustus Brande, *Esq. Apothecary to the Queen.* — — — 38

VI. *Case of a Woman who underwent the Section of the Symphysis Pubis. By Mr.* John Welchman,

Page

Welchman, *Surgeon at Kington in Warwickshire.* — — — 46

VII. *Some Remarks on Hydrocephalus Internus. By Mr.* Edward Ford, *Surgeon of the Westminster General Dispensary.* — 56

VIII. *Some Account of a new Extract of Bark prepared in South America. By* William Saunders, *M. D. Physician to Guy's Hospital.* — — — — 67

IX. *Observations on the Properties commonly attributed by medical Writers to Human Milk, on the Changes it undergoes in Digestion, and the Diseases supposed to originate from this Source in Infancy. By* Joseph Clarke, *M. D. M. R. I. A.— From The Transactions of the Royal Irish Academy, for the Year* 1788. 71

X. *A botanical and medical Account of the Quassia Simaruba, or Tree which produces the Cortex Simaruba. By* William Wright, *M. D. F. R. S. Lond. and Edin. and Physician General in Jamaica.— From The Transactions of the Royal Society of Edinburgh.* 91

Catalogue *of* Books. — 103

